# Views on psychotherapy research among members of the Medical Psychotherapy Faculty of the Royal College of Psychiatrists

**DOI:** 10.1192/bjb.2021.39

**Published:** 2022-04

**Authors:** Marcella Fok, Tennyson Lee, Jessica Yakeley

**Affiliations:** 1 Waterview Personality Disorder Service, Central and North West London NHS Foundation Trust, UK;; 2 Institute of Psychiatry, Psychology and Neuroscience, King's College London, UK; 3 Faculty of Medical Psychotherapy, Royal College of Psychiatrists, London, UK; 4 Deancross Personality Disorder Service, East London NHS Foundation Trust, UK; 5 Centre for Understanding Personality Disorder, London, UK; 6 Portman Clinic, Tavistock and Portman NHS Foundation Trust

**Keywords:** Medical psychotherapy, research, psychotherapy research, academic training, survey

## Abstract

**Aims and method:**

Research drives innovation and improved practice in psychotherapy. We describe views of members of the Faculty of Medical Psychotherapy of the Royal College of Psychiatrists (RCPsych) regarding their knowledge, experience and perspectives on psychotherapy research. We sent questionnaires to the Faculty membership emailing list.

**Results:**

In total, 172 psychiatrists from all levels of training returned fully complete responses. Respondents considered knowledge of psychotherapy research to be important to clinical work. Many have qualifications and experience in research but lack current opportunities for research involvement and would welcome the Faculty doing more to promote psychotherapy research. Perceived obstacles to research involvement included lack of competence, competing demands and wider organisational factors.

**Clinical implications:**

The lack of research opportunities for medical psychotherapists may lead to their underrepresentation in psychotherapy research and a less medically informed research agenda. Providing support at academic, RCPsych and National Health Service organisational levels will allow more clinically relevant research not only in psychotherapy but in other psychiatric disciplines as well.

Research is a cornerstone of psychiatry in all its areas of knowledge, understanding and practice. As a branch of psychiatry, psychotherapy needs research. Although cognitive–behavioural therapy has been considerably researched, the relationship of psychoanalytic psychotherapy to research is complicated. Within psychiatry and among the public, there are common perceptions of psychoanalytic psychotherapy as unscientific and psychotherapists as uninterested in research.

Let us begin by outlining the issue: what is the problem regarding research in psychotherapy? There is evidence that psychoanalytic psychotherapy services within the public sector in the UK have been disproportionately reduced compared with other mental health services.^1^ This may be due to a lack of a robust evidence base in psychoanalytically oriented psychotherapy compared with other modalities, such as cognitive–behavioural therapy. However, high-quality research in psychoanalytic psychotherapy can substantially advance our knowledge of the efficacy of treatment of different mental disorders.^2^ Within the psychoanalytic community a lack of understanding and interest in research persists;^3,4^ this is reinforced by a (real and perceived) split in the community between academics, who do research, and clinicians, who see patients. Many of the trials in psychodynamic psychotherapy have lacked sufficient methodological rigour and do not necessarily reflect real-life practice.^5^

Among psychoanalytic psychotherapists, resistance to research is driven by several factors. Research methods (such as manualisation of treatments, randomisation of patients, recording of sessions, administering outcome measures) are often seen as interfering with clinical technique and practice. Practising therapists may have a poor understanding of the research currency of statistics and numbers and end up finding research activity mindless and meaningless. Moreover, research is treated with suspicion, as it may challenge established and cherished theories.^5^

Nevertheless, there are strong reasons for psychotherapists to engage in research. We need first to investigate the efficacy and outcomes of psychotherapy, to ascertain whether change does occur with treatment, and second to investigate the process of therapy, to understand what happens in therapy and how change occurs. We also need to ensure the safety and quality of treatment and to explore patients’ experiences of psychotherapy. As for commissioners, patients and the public, their expectation of research engagement and the evidence base for psychotherapy needs to be satisfied. Finally, research is an effective means of interrogating new and existing theories and of communicating with colleagues.

One should bear in mind that psychiatric practice involves at its heart the use of a therapeutic relationship, and research into aspects of this relationship can reap useful and highly applicable rewards.^6–8^ The relative sparsity of research in this area reflects the current dominance of the biomedical paradigm within psychiatry. The predominance of cognitive–behavioural therapy, alongside the biomedical paradigm, has played a part in the neglect of psychotherapy research among psychiatrists.

The Faculty of Medical Psychotherapy of the Royal College of Psychiatrists has a membership consisting of psychiatrists who practise psychotherapy as their main therapy or use its principles in their work, as well as psychiatrists who may not be practising psychotherapy but have an interest in the subject. Among those practising psychotherapy, different modalities of therapy may be used; however, psychoanalytic psychotherapy is the main modality in which the majority of medical psychotherapists have been trained. The Faculty is interested in research and is exploring ways of promoting psychotherapy research.

In this study, we surveyed our membership, with the aim of describing psychiatrists’ views on research in psychotherapy and their experiences in engaging with research.

## Method

### Design

A web-based survey was designed by the two joint Research Leads (M.F. and T.L.) of the Faculty of Medical Psychotherapy. The Chair and Vice-Chair of the Faculty were consulted in the design of questions and response options. The College Registrar gave approval to the final version of the survey before its distribution to Faculty members. Survey responses were anonymous.

The survey consisted of 26 questions and covered the following areas:

- general respondent characteristics (member group, current post, work location)
- views on the importance of psychotherapy research
- ways of learning about psychotherapy research
- satisfaction with their own knowledge of psychotherapy research and interest in gaining knowledge
- research experience
- opportunities for, and obstacles to involvement in, psychotherapy research
- views on the Faculty's interest and involvement in research
- suggestions to the Faculty regarding promoting psychotherapy research
- suggestions to support trainees in psychotherapy research.

The format of the questions varied (Appendix) and some questions asked for additional free-text responses. The survey was written and hosted on SurveyHero and was sent out via email to the entire Faculty membership (3842 UK members and 827 overseas members). The email contained a brief message introducing the survey and the reason for doing it, and a direct link to the survey webpage. The email was sent in August 2019 by the College Faculty and Committee Manager. A reminder email was sent before closure at the end of September 2019.

### Participants

All Faculty members, including psychiatrists at all levels of training and experience, were emailed about the survey.

### Analysis

Only fully completed survey responses were included in the analysis. Data were examined numerically and we also identified key themes in the free-text responses.

## Results

We emailed 4669 Faculty members about the survey; 501 persons viewed the survey, and 246 responses were received before the closure date, of which 172 were fully completed responses (i.e. all survey questions answered). The participation rate (number responded out of number viewed) was 49.1% and completion rate (number completed out of number participated) was 69.9%. The response rate (number of responses out of number who were emailed about the survey) was 5.2%. The characteristics of the ‘completed’ respondents are shown in Table 1.

**Table 1:** Characteristics and responses for the ‘completed’ respondents

	Total (*n* = 172)	Consultant with CCT in medical psychotherapy (*n* = 42)	Consultant in other specialty (*n* = 60)	Higher trainee in medical psychotherapy or dual training incl. medical psychotherapy (*n* = 14)	Core trainee or higher trainee in other specialty (*n* = 25)	SAS^a^ or other (*n* = 31)
Medical psychotherapy sessions form part of current post						
Yes	96 (56%)	34 (81%)	24 (40%)	12 (86%)	15 (60%)	11 (35%)
No	76 (44%)	8 (19%)	36 (60%)	2 (14%)	10 (40%)	20 (65%)
Work base						
UK	154 (90%)	41	50 (83%)	13	24	26 (84%)
Outside UK	18 (10%)	1	10 (17%)	1	1	5 16%)
Do you agree that knowledge of psychotherapy research is important for your work?						
Strongly agree	109 (63%)	29 (69%)	36 (60%)	10 (71%)	13 (52%)	21 (68%)
Agree	59 (34%)	12 (29%)	22 (37%)	4 (29%)	11 (44%)	10 (32%)
Neither agree nor disagree	2 (1%)	0	2 (3%)	0	0	0
Disagree	2 (1%)	1 (2%)	0	0	1 (4%)	0
Strongly disagree	0	0	0	0	0	0
Satisfaction with own level of knowledge of psychotherapy research						
Very satisfied	9 (5%)	4 (10%)	1 (2%)	0	0	4 (13%)
Somewhat satisfied	57 (33%)	17 (40%)	21 (35%)	4 (29%)	4 (16%)	11 (35%)
Neither satisfied nor dissatisfied	48 (28%)	12 (29%)	20 (33%)	2 (14%)	4 (16%)	10 (32%)
Somewhat dissatisfied	46 (27%)	8 (19%)	15 (25%)	6 (43%)	13 (52%)	4 (13%)
Very dissatisfied	12 (7%)	1 (2%)	3 (5%)	2 (14%)	4 (16%)	2 (6%)
Formal qualifications in research						
None	90 (52%)	18 (43%)	28 (47%)	9 (64%)	15 (60%)	20 (65%)
BSc	22 (13%)	5 (12%)	9 (15%)	1 (7%)	6 (24%)	1 (3%)
Masters level	31 (18%)	12 (29%)	10 (17%)	4 (29%)	2 (8%)	3 (10%)
Doctorate (PhD/MD)	29 (17%)	7 (17%)	14 (23%)	0	3 (12%)	5 (16%)
Other	13 (8%)	3 (7%)	4 (7%)	0	1 (4%)	5 (16%)
Has ever held paid research post						
Yes	55 (32%)	14 (33%)	23 (38%)	3 (21%)	4 (16%)	11 (35%)
No	117 (68%)	28 (67%)	37 (62%)	11 (79%)	21 (84%)	20 (65%)
Has published non-psychotherapy research						
Yes	97 (56%)	27 (64%)	34 (57%)	3 (21%)	13 (52%)	20 (65%)
No	75 (44%)	15 (36%)	26 (43%)	11 (79%)	12 (48%)	11 (35%)
Has published psychotherapy research						
Yes	50 (29%)	19 (45%)	17 (28%)	0	1 (4%)	13 (42%)
No	122 (71%)	23 (55%)	43 (72%)	14 (1000%)	24 (96%)	18 (58%)
Has current opportunities for involvement in psychotherapy research						
Yes	39 (23%)	12 (29%)	12 (20%)	6 (43%)	4 (16%)	5 (16%)
No	131 (76%)	29 (69%)	48 (80%)	8 (57%)	21 (84%)	25 (81%)
Blank	2 (0%)	1 (2%)	0			1 (3%)
Satisfaction with current opportunities for involvement in psychotherapy research						
Very satisfied	15 (9%)	4 (10%)	4 (7%)	0	0	7 (23%)
Somewhat satisfied	16 (9%)	10 (24%)	1 (2%)	1 (7%)	2 (8%)	2 (6%)
Neither satisfied nor dissatisfied	71 (41%)	15 (36%)	29 (48%)	4 (29%)	9 (36%)	14 (45%)
Somewhat dissatisfied	45 (26%)	10 (24%)	15 (25%)	7 (50%)	8 (32%)	5 (16%)
Very dissatisfied	25 (15%)	3 (7%)	11 (18%)	2 (14%)	6 (24%)	3 (10%)
Perceives obstacles to getting more involved in psychotherapy research						
No	37 (22%)	12 (29%)	9 (15%)	4 (29%)	4 (16%)	8 (26%)
Yes	135 (78%)	30 (71%)	51 (85%)	10 (71%)	21 (84%)	23 (74%)
In your opinion, is the Faculty of Medical Psychotherapy adequately interested and involved in research?						
No	37 (22%)	24 (57%)	12 (20%)	9 (64%)	20 (80%)	2 (6%)
Yes	15 (9%)	3 (7%)	6 (10%)	0 (0)	2 (3%)	4 (13%)
Unsure	120 (70%)	15 (36%)	42 (70%)	5 (36%)	3 (12%)	25 (81%)
Would you like the Faculty of Medical Psychotherapy to do more to promote psychotherapy research?						
No	2 (1%)	1 (2%)	1 (2%)	0 (0)	0 (0)	0
Yes	137 (80%)	34 (81%)	47 (78%)	11 (79%)	20 (80%)	25 (81%)
Unsure	33 (19%)	7 (17%)	12 (20%)	3 (21%)	5 (20%)	6 (19%)

CCT, Certificate of Completion of Training; SAS: Specialist and Associate Specialist doctor; incl., including.

### Respondent characteristics

Of the 172 respondents, 18 (10%) were from outside the UK and the rest were from within the UK; 42 (24%) were consultant psychiatrists with a Certificate of Completion of Training (CCT) in Medical Psychotherapy; 60 (35%) were consultant psychiatrists of other specialties; 14 (8%) were higher trainees in medical psychotherapy (including those in dual training); 25 (15%) were core or higher trainees in other specialties; and 31 (18%) were ‘SAS (Specialist and Associate Specialist) or other’ psychiatrists. Ninety-six respondents (56%) had medical psychotherapy sessions as part of their current post. In terms of research backgrounds, 82 of the respondents (48%) had some research qualification (i.e. BSc, Masters or Doctorate level degree, or other, or a combination of these); 97 (56%) had published non-psychotherapy research; 50 (29%) had published psychotherapy research; and 44 (26%) had published both types of research.

### Views and knowledge of psychotherapy research

When asked ‘Do you agree that knowledge of research is important for your work?’, 168 respondents (97%) answered in the affirmative (‘agree’ or ‘strongly agree’). Respondents were asked to rate their satisfaction with their own level of knowledge in psychotherapy research. Those who had the highest level of satisfaction were consultants in medical psychotherapy (50% were ‘somewhat’ or ‘very satisfied’), followed by SAS or other psychiatrists (48%), consultants in other specialties (37%) and higher trainees in medical psychotherapy (29%). Core and higher trainees in other specialties had the lowest satisfaction rate (16%). Rates of dissatisfaction (i.e. responses ‘somewhat’ or ‘very dissatisfied) ranked almost in the reverse – highest among core and higher trainees in other specialties (68%), followed by higher trainees in medical psychotherapy (57%), consultants in other specialties (27%), consultants in medical psychotherapy (21%) and SAS or other psychiatrists (19%).

Respondents were asked to report which method(s) they used (from five given options and an option ‘other’) to gain knowledge in psychotherapy research. The most common methods were attending conferences (79% of respondents) and reading journals (78%), followed by discussion with colleagues (69%) and using electronic resources (such as saved Google scholar searches) (53%); 20% endorsed ‘involvement in psychotherapy research activity’ as a way of gaining knowledge; 11% reported ‘other’.

### Research experience

Regarding experience in specific research activities, the most common activities were literature review, data collection, and data cleaning or analysis (each reported by 75% of respondents). Also fairly common were writing papers (67%), study design or protocol writing (60%) and recruiting research participants (53%). In total, 38% of respondents had been involved in peer reviewing and 33% in delivering interventions in a trial. Only 3% reported no involvement in any of these research activities.

### Opportunities for psychotherapy research

Thirty-nine respondents (23%) reported having current opportunities for involvement in psychotherapy research – these respondents came from all five member groups (12 consultants in other specialties; 12 consultants in medical psychotherapy; 4 core and higher trainees in other specialties; 6 higher trainees in medical psychotherapy; 5 other psychiatrists).

On rating their current opportunities for involvement in psychotherapy research, 71 respondents (41%) were neutral (neither satisfied nor dissatisfied), 45 (26%) were somewhat dissatisfied and 25 (15%) were very dissatisfied. Fewer respondents were somewhat satisfied (*n* = 16; 9%) or very satisfied (*n* = 15; 9%). Trainees reported higher levels of dissatisfaction (i.e. either somewhat or very dissatisfied: 14 (56%) core and higher trainees in other specialties and 9 (64%) higher trainees in medical psychotherapy) than did non-trainee groups (26 (43%) consultants in other specialties, 13 (31%) consultants in medical psychotherapy, 8 (26%) other psychiatrists).

### Obstacles to involvement in psychotherapy research

The majority of respondents perceived obstacles to becoming involved in psychotherapy research (*n* = 135; 78%). Additional free-text responses to this question were coded and assessed to identify specific themes. The themes identified are shown in Table 2 and the following selection of free-text responses.

**Table 2:** Perceived obstacles to getting more involved in psychotherapy research

Age/retirement
Clinical workload
Lack of contacts or potential collaborators
Lack of funding/infrastructure/research administrative support
Lack of knowledge/competence/confidence
Lack of opportunities
Lack of personal interest
Lack of senior colleague support/mentoring
Lack of time/competing interests or commitments
Not in research post or no allocated time in job plan
Wider organisational factors
Other

Lack of time, competing demands:

‘Dedicated research time has been removed from my job plan. Clinical and managerial pressures now make research very difficult.’

‘Mainly lack of dedicated time and links with established psychotherapy researchers.’

Lack of support and contacts:

‘Too little time; no admin support for the scout work; no team or group to support applications; hostile competition from psychology and psychiatry; hopeless stereotypes about medical psychotherapy.’

‘Support and time. It requires membership of a group. I have not been able to develop these in spite of trying to collaborate with research psychologists.’

Lack of opportunities, wider organisational factors:

‘Don't know who to contact/not aware of any current psychotherapy research projects being undertaken within my trust/its associated academic institute.’

‘There simply is no psychotherapy research as far as I know.’

‘Not seen as a priority by academics, therefore not encouraged/supported.’

‘No good research going on – multicentre – in my area of interest that is psychodynamic.’

‘There just isn't a lot going on and when I do find some to be involved in it's hard to get my name on the paper if and when it gets published.’

Lack of potential collaborators, lack of senior colleague support:

‘The lack of psychotherapy research that I would be interested in in close enough proximity to where I work.’

‘Limited interest in research among colleagues and trainers.’

‘The high-flying research department I work in regularly shunned psychotherapy research related proposals I made for seven years.’

### Faculty role and activities to promote psychotherapy research

The majority of respondents (*n* = 120, 70%) were unsure whether the Faculty of Medical Psychotherapy was adequately interested and involved in research, 22% (*n* = 37) felt that it was not and 9% (*n* = 15) felt that it was. However, most respondents (*n* = 137, 80%) said they would like the Faculty to do more to promote psychotherapy research. Many (*n* = 125, 73%) said they would be interested in participating in Faculty activities to do with psychotherapy research. Respondents were asked what they would like the Faculty to do; they were offered six options, from which they could select as many as they wished (Table 3). The most popular option was ‘Facilitate networking among members who are interested or involved in research’. Additional free text responses gave further ideas:

‘Ask the College to help make links with academics and possible sources of funding.’

‘Identify research experts.’

‘Link with other established research bodies.’

‘Network with other faculties, their newsletters, identify gaps and encourage joint working in projects.’

‘Networking could extend to mentoring.’

‘Pair trainees with research-orientated psychotherapists to inculcate a culture of research in next generation of psychotherapists.’

‘The Faculty could argue for the return of one day per week for research and the completion of the equivalent of an MSc in research.’

**Table 3:** Interventions the Faculty should deliver to promote psychotherapy research (*n* = 172; multiple selections allowed)

Facilitate networking among members who are interested or involved in research	132 (77%)
Feature articles related to research in the Faculty newsletter or other communication	119 (69%)
Offer conferences on psychotherapy research	118 (69%)
Organise skills workshops or webinars on research methodology	116 (67%)
Compile practical tips and guidance for setting up research projects	115 (67%)
Make psychotherapy research journals more accessible to members (e.g. via RCPsych library services)	104 (60%)
Other	12 (7%)

### Trainees and psychotherapy research

When asked what the Faculty could do to specifically support trainees to get involved in psychotherapy research, the most popular response (of the four options offered), among both trainees and non-trainees, was ‘Help link up psychotherapy research supervisors to trainees’ (voted by *n* = 148 (86%) respondents). ‘Offer small grants, or a trainee award or prize for psychotherapy research’ and ‘Place more emphasis on research within the psychotherapy curriculum’ were voted by *n* = 108 (63%) and *n* = 90 (52%) respondents respectively. Again, free-text responses gave further elaborations and ideas, such as the following.

Placing research on the training agenda:

‘There should be a better balance of what is asked of us within the curriculum. The more we get space and time to work with research that interests us during our core training, the more we will be able to continue to do it in the future and make an actual difference in research.’

‘It needs to be valued as a pursuit and encouraged as a part of the career path rather than a defeatist and sometimes elitist attitude precluding most from pursuing it.’

Action from trainers and organisations:

‘Encourage HEE [Health Education England] to develop more research-oriented training posts.’

‘Have psychotherapy consultants promoting a research-oriented practice.’

Senior-level development opportunities:

‘Develop consultants as well as trainees.’

‘It would be good to establish senior academic positions in medical psychotherapy.’

Promoting psychotherapy research:

‘Identify a list of research questions that psychotherapy research would be able to answer and publish it and regularly update it so that trainees can be inspired and if they would like to do research, may consider choosing a topic.’

‘More emphasis on psychotherapy research across all the curricula not just the psychotherapy curriculum.’

Other comments:

‘Not sure. Depends on the amount of time the trainee has. Pursuing the research agenda may be important but it is not as important as obtaining a thorough and secure grounding in clinical psychotherapy.’

## Discussion

### Main findings

In this first ever survey of the membership of the Faculty of Medical Psychotherapy on research, psychiatrists across all levels of training and experience, working within and outside of medical psychotherapy as a specialty, strongly endorsed the importance of knowledge of psychotherapy research in their work. There was a high level of research experience or qualification among the survey respondents – almost half held a research degree, one-third had held a paid research post and 97% had engaged in some kind of research activity.

Given the low response rate to the survey, these findings cannot be taken as representative of the membership of the Faculty in general. Nevertheless, the survey highlights the existence of a group of members within the Faculty who are interested and engaged in research, and gives an indication of how the Faculty can play a part in this area. Despite the high prevalence of research qualifications and experience, only a minority of respondents had current opportunities for involvement in research, and the majority perceived obstacles to engaging in psychotherapy research. This points to an untapped potential and resource for psychotherapy research and begs the question of what one can or should do with it.

### Strengths and limitations

Our survey is the first of its kind for the Faculty and addresses an important issue for training and development in medical psychotherapy. The questions were designed to extract relevant background data, views and experiences that can inform the Faculty's strategy. The entire Faculty membership was surveyed and the low response rate means that the findings cannot be regarded as representative of the Faculty membership at large. The Faculty has a large number of quiescent members and this is also a factor in the low response rate. To put this in context, there were only 269 doctors with medical psychotherapy (or psychotherapy) as their specialty listed on the General Medical Council specialist register in 2019.^9^ This indicates that we had 42/269 (16%) of specialty-listed medical psychotherapists responding in this survey. Many of these doctors may not be working in designated psychotherapy posts or be practising psychotherapy. The number of doctors in postgraduate training in medical psychotherapy in the UK in the same year was 37. This indicates we had 14/37 (38%) of medical psychotherapy trainees responding.

One expects that members who are more research-inclined were more likely to take time to respond to the survey, thus biasing the results towards a more pro-research direction (i.e. viewing research as more important and having greater experience and interest in research) than would be found across the membership in general. Likewise, the views on research opportunities or lack thereof, and desire for more Faculty engagement with research, cannot be generalised across the entire membership of the Faculty. The responses may be subject to some degree of bias due to social desirability, although the free-text responses suggested considerable frankness of expressed views. The choice of interventions that the Faculty could deliver to promote research and support trainees in research were based on a pre-determined list of options and may not have covered all possibilities.

Nevertheless, the survey highlights the presence of a group of research-inclined members in the Faculty and points to ways that these members can be helped to participate more actively in research. Members are keen for the Faculty to facilitate networking. This may mitigate against the sense of isolation and disconnection that individuals may face among local colleagues or within organisations with little interest in psychotherapy research. Networking can take a number of forms – for example connecting experts and supervisors with trainees, linking with other faculties (such as the Faculty of Academic Psychiatry), links with established research and funding bodies. Other ideas for the Faculty to implement include featuring articles on research more prominently in newsletters, organising academic activities (e.g. conferences, skills workshops) on research, offering practical guidance on setting up projects, and better access to psychotherapy research journals (Box 1 lists useful resources on research).

Box 1 Useful resources related to research

Publications

- Davis WE, Giner-Sorolla R, Lindsay DS, Lougheed JP, Makel MC, Meier ME, et al. Peer-review guidelines promoting replicability and transparency in psychological science. *Adv Meth Pract Psychol Sci* 2018; **1**: 556–73.
- Rhodes M. How to undertake a research project and write a scientific paper. *Ann R Coll Surg Engl* 2012; **94**, 297–9.

Online guidelines

- Planning a good research project (Postgrad.com): https://www.postgrad.com/uk_research_planning/
- Basic steps in the research process (North Hennepin Community College): https://www.nhcc.edu/student-resources/library/doinglibraryresearch/basic-steps-in-the-research-process
- Policies and guidance for researchers (UK Research and Innovation): https://mrc.ukri.org/research/policies-and-guidance-for-researchers/#policies
- Guidelines for completing a research protocol for observational studies (University College London Hospitals): http://www.sld.cu/galerias/pdf/sitios/revsalud/guidelines_for_observational_studies.pdf

Structural and organisational issues were also highlighted in the survey responses. The real and perceived disinterest and even hostility of academic institutions towards psychotherapy research, sometimes combined with negative preconceptions about medical psychotherapy, especially psychoanalytically oriented psychotherapy, create a culture that does not consider it possible for medical psychotherapists to engage in research. To an extent, this is reflected in certain deficits in research academic development opportunities for medical psychotherapy. In England, the National Institute for Health Research (NIHR) Integrated Academic Training Programme provides academic opportunities for doctors and dentists in specialty training, through the funding of Academic Clinical Fellowship (ACF) and Clinical Lectureship (CL) posts that support trainees to spend 25% (in the case of ACF) or 50% (CL) of their time in research training over 3 or 4 years. In more than a decade of this programme, no single trainee has been awarded such a post within the specialty of medical psychotherapy.

There are top-down as well as bottom-up problems to be addressed. The historical lack of research-active senior medical psychotherapists and the absence of medical psychotherapists within academic institutions means that medical psychotherapy has become a non-existent entity in many research circles. Some argue that research should be more embedded in training in medical psychotherapy, in which the prevailing emphasis is on acquiring clinical psychotherapeutic skill; they believe that knowledge of psychotherapy research, routine use of clinical outcomes, and experience in designing and conducting research should all form part of the curriculum for trainees. Among medical psychotherapy trainees, designated time that is meant for research or special interest (such sessions exist for psychiatry trainees of all specialties) is often used instead for further clinical experience or for personal psychotherapy.

Senior and consultant-level medical psychotherapists in the public sector have faced increasing cuts to sessions and their job plans focus exclusively on clinical service delivery; this approach is short-sighted and deprives medical psychotherapy of possibilities for development. A more beneficial strategy would be to encourage those medical psychotherapists with research experience and interests to pursue projects as part of their job plan, and to provide support where needed to facilitate this. Where a National Health Service (NHS) organisation already has an established partnership with an academic institution, links for psychotherapy research can be set up and formally endorsed by both organisations. Previous research has indicated that, to be willing to participate in research, psychotherapists expected high-quality designs, financial compensation and personal gains.^10^ This indicates the importance of support to therapists at both research and career-progression levels to encourage more participation in research.

## Conclusions

From the survey, we conclude that there exists a group of members within the Faculty of Medical Psychotherapy who value research and are keen to engage in research activity, and are a resource that ought to be harnessed. Noting the greater level of dissatisfaction with their research involvement among trainees than among consultants, this is a particularly important group to focus resources on. A number of actions recommended by survey respondents are feasible and already being planned, for example establishing research networks, linking trainees with supervisors, conferences and workshops on research, and campaigning for more equitable academic opportunities nationally. Given the role of medical psychotherapists in combining a medical, psychiatric and psychotherapeutic perspective,^11^ it is critically important that this unique perspective is brought to bear on the psychotherapy research agenda.

## Data Availability

The data that support the findings of this study are available from the corresponding author, M.F., upon reasonable request.

## About the authors

**Marcella Fok** is a consultant psychiatrist in psychotherapy for the Waterview Centre, a specialist personality disorder service within Central and North West London NHS Foundation Trust, and a visiting researcher at the Institute of Psychiatry, Psychology and Neuroscience, King's College London, UK. **Tennyson Lee** is clinical lead at Deancross Personality Disorder Service, East London NHS Foundation Trust, and co-director of the Centre for Understanding Personality Disorder (CUSP), London, UK. Marcella Fok and Tennyson Lee are joint Research Leads for the Faculty of Medical Psychotherapy, Royal College of Psychiatrists, UK. **Jessica Yakeley** is current Vice-Chair and previous Research Lead for the Faculty of Medical Psychotherapy, Royal College of Psychiatrists, UK; she is a consultant psychiatrist in forensic psychotherapy and Director of the Portman Clinic, and Director of Medical Education at the Tavistock and Portman NHS Foundation Trust, London, UK.

## Appendix 1

Survey questions

1. Are you a member of the Medical Psychotherapy Faculty of the RCPsych? If not, this survey is not for you.
2. Are you a:
  - Consultant with CCT in Medical Psychotherapy; Higher Trainee in Medical Psychotherapy (or dual training including medical psychotherapy); Consultant in other specialty (please specify); Core Trainee or Higher Trainee in other specialty (please specify); Other (please specify)
3. Is your work base in the UK?
  - UK; outside of UK. Please specify the geographical region or area
4. What is your current post? Please enter (e.g. consultant in eating disorders)
5. In your current post, do you have any sessions in medical psychotherapy? Please give details if you wish
6. Do you agree that knowledge of psychotherapy research is important for your work?
  - Strongly agree; Agree; Neither agree nor disagree; Disagree; Strongly disagree
7. In which aspect of your work would you most like more knowledge of existent psychotherapy research?
8. How do you gain knowledge of psychotherapy research? (tick all that apply)
  - Reading journals; Using electronic resources (e.g. email alerts, saved scholar searches); Discussion with colleagues; Attending conferences; Involvement in psychotherapy research activity (please specify); Other (please specify)
9. Are you satisfied with your current level of knowledge in psychotherapy research?
  - Very satisfied; Somewhat satisfied; Neither satisfied nor dissatisfied; Somewhat dissatisfied; Very dissatisfied
10. Have you any formal qualifications in research? (please tick all that apply)
  - None; BSc, MSc, PhD or MD equivalent; Other (please specify)
11. Have you ever held a paid research post? Please specify
12. Have you ever been involved in the following kinds of research (not necessarily psychotherapy related)?
  - Qualitative, Quantitative, Neither; Observational, Experimental, Neither; Epidemiological, Outcome, Neither
13. What research activities have you ever been involved in? (please tick all that apply)
  - Literature review; Study design or protocol writing; Recruiting research participants; Delivering intervention in a trial; Data collection, cleaning and/or analysis; Paper writing; Peer reviewing; Other (please specify); None
14. Have you ever published non-psychotherapy research in a peer-reviewed journal?
15. Have you ever published psychotherapy research in a peer-reviewed journal?
16. Currently, do you have any opportunities for involvement in psychotherapy research? Please specify
17. Are you satisfied with your current opportunities for involvement in psychotherapy research?
  - Very satisfied; Somewhat satisfied; Neither satisfied nor dissatisfied; Somewhat dissatisfied; Very dissatisfied
18. Do you perceive obstacles to getting more involved in psychotherapy research? If yes, please specify
19. In your opinion, is the Medical Psychotherapy Faculty adequately interested and involved in research?
  - Yes; No; Unsure. Please give reason for your answer
20. Would you like the Medical Psychotherapy Faculty to do more to promote psychotherapy research?
  - Yes; No; Unsure. Please give reason for your answer.
21. What kinds of things should the Faculty do to promote psychotherapy research? (please tick all that apply) [The list of options appears in Table 3 of this paper]
22. What can the Faculty do to specifically support trainees to get involved in psychotherapy research? (please tick all that apply)
  - Place more emphasis on research within the psychotherapy curriculum; Link up psychotherapy research project supervisors to trainees; Offer small grants, or a trainee award or prize for psychotherapy research; Other (please specify)
23. Are you interested in participating in Faculty activities to do with psychotherapy research?
  - Extremely interested; Very interested; Somewhat interested; Not so interested; Not at all interested. Please leave your name and email and say something about your specific interest
24. Please leave any further comments you have on this subject here
