# Views on psychotherapy research amongst members of the Medical Psychotherapy Faculty of the Royal College of Psychiatrists – CORRIGENDUM

**DOI:** 10.1192/bjb.2021.74

**Published:** 2022-08

**Authors:** Marcella Fok, Tennyson Lee, Jessica Yakeley

**Keywords:** Medical psychotherapy, research, psychotherapy research, academic training, survey

In the original published article, one of the affiliations for co-author Tennyson Lee was cited incorrectly. This has now been updated in the online published article.

The authors apologise for this error.
